# A pilot study on the effects of a physio-avatar EB experience on motor performance

**DOI:** 10.3389/fbioe.2026.1767062

**Published:** 2026-06-15

**Authors:** Ryoma Kojima, Kazuhiro Matsui, Kotaro Okada, Ruu Fujii, Keita Atsuumi, Yoshiki Mori, Hiroaki Hirai, Atsushi Nishikawa

**Affiliations:** 1 Graduate School of Engineering Science, The University of Osaka, Toyonaka, Japan; 2 Faculty of Information Science and Arts, Osaka Electro-Communication University, Shijonawate, Japan; 3 Graduate School of Information Sciences, Hiroshima City University, Hiroshima, Japan

**Keywords:** avatar embodiment, electromyography, internal models, motor performance, neurorehabilitation, sense of agency, sense of ownership, virtual reality

## Abstract

**Introduction:**

Neurorehabilitation using virtual reality (VR) and electromyography has garnered attention in recent years. The authors developed a system based on these technologies that enables participants to experience an avatar (physio-avatar EMG biofeedback, PAEB) with arbitrary dynamics in VR environments, controlled by their own EMG signals. Using this system, the study examined whether experiencing an avatar that feels slower than the real body can promote the relearning of cerebellar *internal models* and improve motor performance.

**Methods:**

Experiments were conducted in 30 healthy adults. Participants were assigned to four groups: the PAEB adaptation group (n = 6), which underwent prolonged PAEB training; the PAEB nonadaptation group (n = 7), which received only short-term PAEB exposure; the control group (n = 8), which experienced a real-body avatar (RBA) reflecting actual movements without EMG control; and the resistance training (RT) control group (n = 9), which performed isometric contractions comparable to the PAEB adaptation group without visual avatar feedback. Motor performance was evaluated based on angular velocity during a real-body reaching task. Each group performed the task before and after the adaptation phase to assess the effects of PAEB exposure. Subjective questionnaires were administered to evaluate the sense of ownership (SoO) and sense of agency (SoA).

**Results:**

Subject-level one-tailed Mann-Whitney U (α=0.05) indicated significant improvements in motor performance in five of six participants in the PAEB adaptation group. In contrast, no significant improvements were observed in the other groups, except for one participant in the PAEB nonadaptation group and one participant in the control group and one participant in the RT control group. Notably, no participant in the RT control group showed a significant improvement; instead, most exhibited a decrease in angular velocity. For within-group comparisons, the one-tailed Wilcoxon signed-rank test (α=0.05) showed that only the PAEB adaptation group demonstrated a substantial increase (25.4%) in angular velocity from baseline to post-adaptation. Questionnaire results further indicated that only the PAEB adaptation group showed a tendency toward increased SoA with repeated PAEB exposure.

**Discussion:**

The improvement in motor performance observed in the PAEB adaptation group suggests that exposure to an avatar with altered bodily dynamics modifies motor control strategies through error-driven updating of internal models. This interpretation is supported by the RT control group, which showed no improvement despite experiencing comparable physical load. These findings indicate that the enhancement in motor speed is more likely attributable to internal model recalibration via error learning than to acute neuromuscular responses to physical training.

**Conclusion:**

These results indicate that experiencing the PAEB–an avatar that appears to move slower than one’s own body–can potentially improve motor performance. However, as the number of participants who underwent PAEB training was limited, further studies with a larger sample size are required to confirm these findings.

## 1 Introduction

Human motor control relies on *internal models* constructed within the brain, which provide a neural framework for generating motor commands. The cerebellum holds and updates these internal models based on discrepancies between predicted and actual sensory feedback (Miall and Wolpert, 1996). These internal models are generally categorized into *forward* and *inverse models*. The forward model predicts the sensory consequences of a motor command (e.g., visual, proprioceptive, or tactile sensations), whereas the inverse model computes the motor command required to achieve a desired movement outcome (Kawato, 1999). These models exhibit high plasticity and are modified based on experience. When the actual sensory outcome of a movement deviates from the predicted outcome, the brain performs re-adaptation based on the resulting error. This process, known as *error learning*, is a central concept in modern neuroscience and rehabilitation research (Shadmehr and Krakauer, 2008).

In individuals with neurological impairments, such as stroke or other disorders, this error learning process is often markedly disrupted. Damage to neural pathways can impair the prediction of sensory consequences and the updating of internal models, leading to persistent motor deficits. Consequently, the safe and effective induction of error learning, together with accurate assessment of its integrity, is critical in neurorehabilitation.

Conventional rehabilitation typically relies on repetitive physical exercises to promote motor recovery and on clinical scales to evaluate progress. However, these approaches have several limitations. They can be highly monotonous, reducing patient motivation for sustained therapy (Laver et al., 2017). In addition, conventional assessments are largely subjective and do not enable continuous, quantitative monitoring of underlying motor learning processes, such as real-time adaptation of internal models during task execution.

To address these limitations, interfaces integrating virtual reality (VR) and electromyography (EMG) have garnered considerable attention as novel tools for motor learning. VR systems using head-mounted displays (HMDs) offer safe, flexible, and immersive environments that promote neural plasticity and functional reorganization of the brain (Levin et al., 2015; Aramaki et al., 2019), thereby helping maintain patient motivation. Moreover, these interfaces directly contribute to the objective and quantitative assessment of the motor learning process because they continuously monitor the user’s physiological signals.

EMG-driven interfaces, particularly when integrated with robotic or VR-based rehabilitation systems, represent the current state of the art. For example, EMG-driven robotic rehabilitation enables control based on muscle activity even in the presence of limited voluntary joint movement, thereby promoting active patient engagement in therapy (Huo et al., 2023; Sarhan et al., 2023). Accordingly, rehabilitation systems that integrate EMG and VR have been widely investigated as promising clinical approaches (Toledo-Peral et al., 2022; Lin et al., 2023). The incorporation of VR into EMG-driven systems offers an emerging avenue to enhance patient motivation and sensorimotor feedback, while enabling continuous and objective assessment of motor learning processes.

The authors previously developed a prototype system, termed *physio-avatar EMG biofeedback* (PAEB,/peɪb/), to integrate VR and EMG within the theoretical framework of internal models (Ando et al., 2024). Although prior studies, including preliminary work, suggest that exposure to an EMG-driven avatar with altered bodily dynamics can induce changes in motor control strategies at the level of muscle activity, a critical gap remains. Specifically, it is unclear whether these EMG-level adaptations translate into measurable improvements in objective motor performance, such as movement speed or reaction time.

The primary objective of this study was to determine whether adaptation to the PAEB system influences motor performance. Accordingly, a preliminary validation (pilot) study was conducted in healthy participants. Specifically, the authors assessed whether exposure to the PAEB—an avatar designed to induce sensory prediction errors by moving more slowly than the user’s actual limb—enhances maximum angular velocity during a subsequent real-world reaching task. In addition, changes in the sense of ownership (SoO) and sense of agency (SoA) during the avatar experience were examined to explore their relationship with motor performance.

The remainder of this paper is organized as follows. Section 2 reviews related work, including conventional neurorehabilitation approaches, avatar embodiment, and prior studies on the PAEB system. Section 3 describes the materials and methods, including participant characteristics, experimental setup, task design, and data processing procedures. Section 4 presents the results, covering both subjective questionnaire scores and objective motor performance metrics. Section 5 discusses the findings, and Section 6 concludes the paper with a summary and directions for future research.

## 2 Related works

### 2.1 Conventional neurorehabilitation and robotics

Recent studies on EMG-driven robotic rehabilitation frameworks have highlighted their advantages for rehabilitation. EMG-based robotic systems have been shown to improve upper-extremity motor function in post-stroke patients (Huo et al., 2023). In addition, EMG/EEG-based control schemes enable the estimation of motor intent and real-time control of rehabilitation robots (Sarhan et al., 2023). Although the present study employs an armrest setup—constructed from aluminum frames and equipped with a force sensor—to stabilize the forearm during isometric contractions, rather than an active robotic device (e.g., an actuator-driven exoskeleton), it similarly utilizes real-time physiological signals to infer motor intent and provide visual feedback. Insights from these robotic systems support the potential of the proposed framework to drive motor adaptation through related underlying mechanisms.

### 2.2 Avatar embodiment and motor control

The advancement of VR has facilitated research on physical and cognitive modifications induced by avatar experiences. Previous studies have demonstrated that experiencing a body different from one’s body can significantly alter cognition and perception. For instance, Keizer et al. (2016) demonstrated that inducing a sense of body ownership over an avatar with a different body type could improve body image disturbances in patients with anorexia nervosa. Furthermore, Ratan and Sah (2015) experimentally investigated the stereotype threat effect and found that avatar embodiment could influence cognitive performance, such as math scores. These findings indicate that the effect of avatar experiences extends beyond the virtual space, potentially affecting brain processing. In the context of rehabilitation, inducing a strong SoO and SoA toward an avatar is crucial (Preston and Ehrsson, 2014; Waltemate et al., 2018; Tieri et al., 2017).

### 2.3 Previous studies on physio-avatar EB

Building on these concepts, the authors previously developed a prototype system, termed PAEB, which integrates VR and EMG within the theoretical framework of internal models (Ando et al., 2024). The PAEB system projects an arm-shaped avatar in a first-person VR environment, enabling continuous, real-time control based on the user’s EMG signals via a neuromusculoskeletal system (NMSS) model. Unlike approaches that rely primarily on machine learning or trigger-based control for discrete movements (Yang et al., 2009), the PAEB system supports continuous motion control. Preliminary work in healthy participants showed that exposure to an EMG-driven avatar with altered physical dynamics—specifically, slower response and increased damping relative to the user’s actual limb—induces sensory prediction errors and modulates motor control strategies at the level of muscle activity (Ando et al., 2024). However, this prior study focused solely on EMG-level changes, leaving a critical gap. It remains unclear whether such adaptations translate into measurable improvements in objective motor performance, such as maximum angular velocity during reaching tasks. Addressing this gap is the primary aim of the present study.

## 3 Methods

### 3.1 Participants

Thirty healthy adults (28 males and 2 females; mean age = 22.9±0.96 years) participated in this study. They were randomly allocated to one of four experimental groups:

- PAEB adaptation group (6 males; 22.8±1.06 years)
- PAEB nonadaptation group (6 males and 1 female; 22.7±1.11 years)
- Control group (8 males; 22.6±0.92 years)
- RT control group (8 males and 1 female; 23.3±0.71 years)

The PAEB adaptation group underwent a sufficiently long PAEB experience to ensure full adaptation to the PAEB system. In contrast, the PAEB nonadaptation group underwent a short PAEB session, which was insufficient for full adaptation to the PAEB system. The authors’ previous findings indicated that motor performance did not improve under short exposure to the PAEB system (Kojima et al., 2025). The control group did not undergo the PAEB intervention. The RT control group performed isometric contractions at an intensity equivalent to the PAEB group without any visual avatar feedback, serving as a physical-load-matched control.

The participants were not informed of the specific hypothesis of this study or the expected direction of change, thereby minimizing demand characteristics and unintended bias.

### 3.2 Apparatus and system design

**FIGURE 1:**
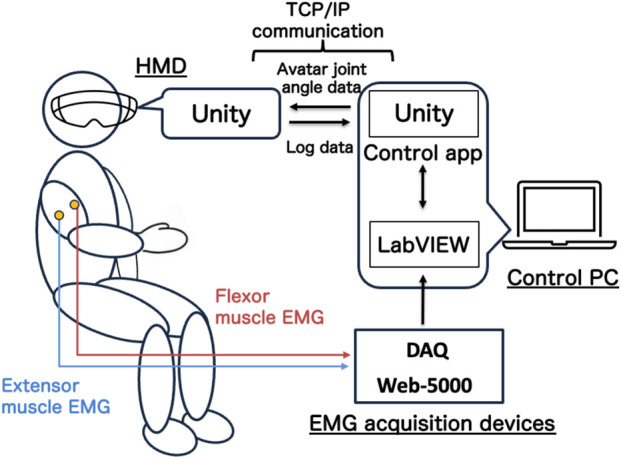
Configuration of the PAEB system. Note: EMG signals obtained from the flexor and extensor muscles are processed through the EMG acquisition device and LabVIEW to compute the joint angle of the avatar. These joint-angle data are transmitted from LabVIEW to the control application (Unity) via internal TCP/IP communication within the PC. The control application then transmits these data to the HMD via TCP/IP communication, updating the avatar’s motion in real-time. The HMD transmits the avatar’s joint-angle log data back to the control application via TCP/IP communication.

A modified version of the PAEB prototype developed by Ando et al. was used, as shown in Figure 1 (Ando et al., 2024). This system contained two main components:

- Display unit: Meta Quest 3 (Meta Platforms, Inc.)
- Computation unit: LabVIEW 2024 (NI), EMG amplifier device WEB-5000 (NIHON KOHDEN CORPORATION), and A/D converter USB-6363 (NI)

Surface EMG signals were collected from the biceps brachii (flexor) and triceps brachii (extensor) using wet electrodes at a sampling rate of 1,000 Hz. A two-point calibration method was used to consider the variations in the EMG signal amplitude. As the relationship between EMG and tension can become nonlinear during high-intensity contractions (Lawrence and De Luca, 1983), normalization based on maximum voluntary contraction (MVC) was not performed. Instead, the signals were normalized using two points: EMG voltages obtained during relaxation and when participants maintained a steady, sub-maximal contraction (verbally instructed as “approximately 80% effort”). The processed EMG data were converted into a single-degree-of-freedom right-elbow joint angle using the neuromusculoskeletal system (NMSS) model (Ando et al., 2024). The joint-angle values computed in LabVIEW were transmitted via internal TCP/IP to the *control application*, which relayed the data to the HMD (also via TCP/IP communication) to update the avatar’s motion in real-time.

The HMD operated at a refresh rate of 90 Hz with 2064×2208 pixels per eye, and joint-angle updates were performed at 100 Hz.

**FIGURE 2:**
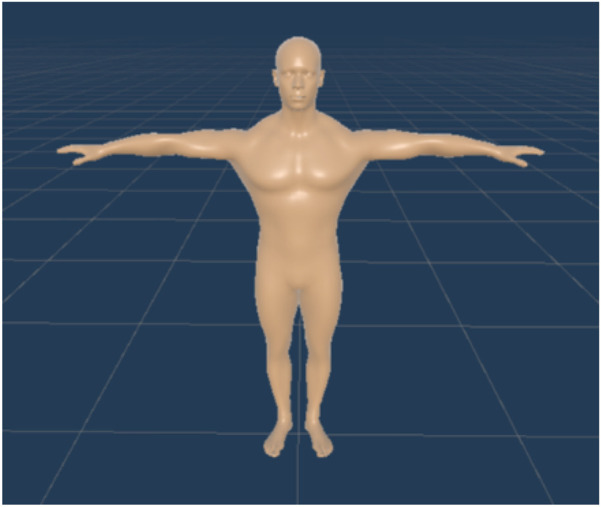
Avatar. Note: Appearance of the VRM avatar. The VRM format is a 3D model specification with a humanoid skeletal structure.

A humanoid avatar was displayed in the virtual environment, as shown in Figure 2. A VRM-format avatar with a humanoid skeletal structure was employed during the experiment. The VRM format is a standardized 3D humanoid model specification based on glTF 2.0 and can be controlled on Unity via the UniVRM package (VRM Consortium, 2023; VRM Consortium, 2024). The upper-arm and forearm lengths of each participant were measured before the experiment, and avatars were created to match the participant’s body dimensions.

Two types of avatar control were implemented:

1. Real-body avatar (RBA), which reproduced the participant’s actual movements. The avatar motion was synchronized with participants’ body movement using the depth sensor of the HMD, enabling the avatar to mirror the participants.
2. PAEB, in which the avatar’s dynamics was modified by changing the parameters of the NMSS model.

**FIGURE 3:**
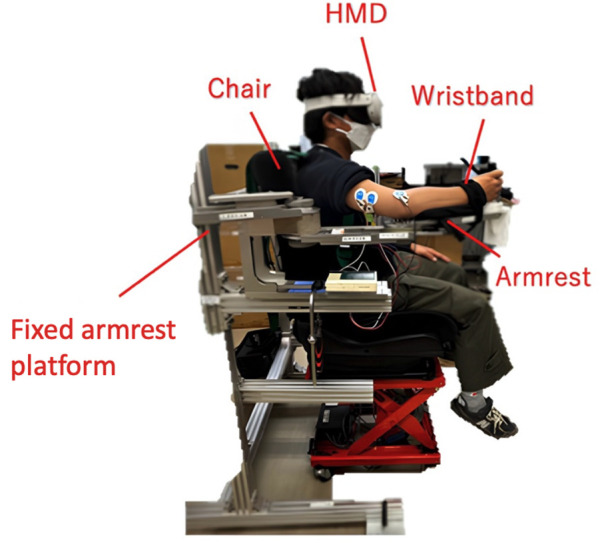
Posture of participants while experiencing the proposed system. Note: The participants experienced the proposed PAEB system while seated on a fixed chair. The image shows an example posture under the PAEB condition. The RT control group maintained the same posture, except that EMG electrodes and wristbands were not used.

The participants maintained the posture shown in Figure 3 in the experimental environment. They sat on a fixed chair and rested the right elbow on an armrest, which served as the baseline posture. In the PAEB and RT conditions, the distal part of the forearm was secured to the armrest to restrict movement to ensure isometric contraction. In the RT condition, a six-axis force sensor (CFS034CA501U, Leptrino) mounted on the armrest was used to measure force output. Sensor signals were sampled at 1,000 Hz and used to provide real-time visual feedback of force magnitude and monitor exertion intensity. Force levels were calibrated in advance by instructing participants to exert approximately 60% of their maximum effort. In the RBA mode, the wrist was not fixed and the forearm was allowed to move freely while the elbow remained on the armrest. The armrest height was adjusted individually such that each participant maintained a comfortable and natural posture.

### 3.3 Data processing

The collected surface EMG signals were processed to compute the input variables for the NMSS model. To eliminate power-line interference noise, a hardware hum filter (60 Hz notch filter) built into the WEB-5000 was applied to the raw EMG signals. Additionally, the signals were processed using a hardware band-pass filter (16–100 Hz), followed by a software low-pass filter (100 Hz), full-wave rectification, and a final software low-pass filter (22 Hz) implemented in LabVIEW. The 16–100 Hz hardware band-pass setting was selected as the available WEB-5000 device setting closest to the 20–100 Hz band-pass range used in a prior study (Bhagat et al., 2014). The final 22 Hz low-pass filter after rectification was selected as a smoothing cutoff close to the 24 Hz low-pass filter used in a prior study (Gazendam and Hof, 2007).

Elbow joint kinematics were recorded as a continuous time series. Angular velocity was computed by first applying spline interpolation to the angle data, followed by smoothing with a moving average filter to suppress high-frequency noise introduced by differentiation, and subsequent numerical differentiation. The peak value within the movement interval of each trial was then extracted from the resulting angular velocity profile as the maximum angular velocity.

### 3.4 Parameter settings of the NMSS model

The NMSS model is a computational model that linearly approximates the dynamics of the NMSS to estimate virtual joint angles (Ando et al., 2024; Matsui et al., 2014; 2022; Suzuki et al., 2024; Hori et al., 2025). It integrates the transfer functions of the neuromuscular system (NMS) and musculoskeletal system (MSS), which are represented by Equations 1 and 2, respectively (Ando et al., 2024). In these equations, $G_{\text{NM}}$ and $G_{\text{MS}}$ denote the transfer functions of the NMS and MSS, respectively; K is the system gain; $\omega_{\text{n}}$ is the natural angular frequency; ζ is the damping ratio; and L denotes the time delay. The subscripts NM and MS correspond to the neuromuscular and musculoskeletal systems, respectively.

$$G_{\text{NM}}\left(s\right)=\frac{K_{\text{NM}}\omega_{\text{nNM}}^{2}}{s^{2}+2\zeta_{\text{NM}}\omega_{\text{nNM}}s+\omega_{\text{nNM}}^{2}}e^{-L_{\text{NM}}s}$$ (1)

$$G_{\text{MS}}\left(s\right)=\frac{K_{\text{MS}}\omega_{\text{nMS}}^{2}}{s^{2}+2\zeta_{\text{MS}}\omega_{\text{nMS}}s+\omega_{\text{nMS}}^{2}}$$ (2)

The parameters were set to $K_{\text{NM}}\cdot K_{\text{MS}}=500$ , $\omega_{\text{nNM}}=\omega_{\text{nMS}}=5.0\;\left[rad/s\right]$ , and $\zeta_{\text{NM}}=\zeta_{\text{MS}}=0.9$ . With these parameter settings, the avatar’s motion exhibited slower and more damped responses than the participant’s actual movement; this resulted in a subjective sensation of difficulty in movement (Okamoto et al., 2024). To prevent cybersickness caused by an excessive mismatch between the real and virtual bodies (Suglia et al., 2023; Winter et al., 2021; Serge and Fragomeni, 2017), the parameters were carefully adjusted during the design phase, thereby making the avatar’s movement delay perceptible rather than excessive. This manipulation introduced sensorimotor discrepancies, thereby promoting the re-adaptation of the internal models and improving the motor speed via error learning.

### 3.5 Task design

The experimental tasks comprised three major components: the *reaching task*, the *adaptation task*, and the *washout task*.

**FIGURE 4:**
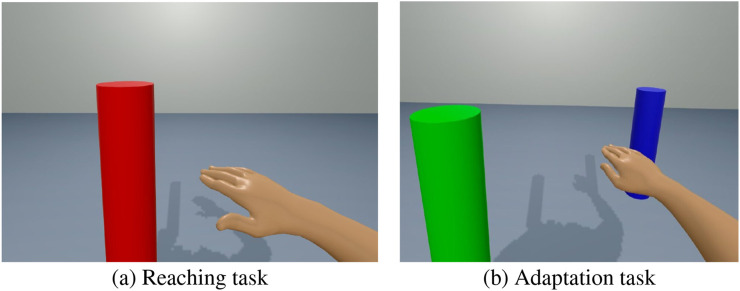
Examples of reaching and adaptation tasks. Note: **(a)** The target object was placed at a position reachable by 60° elbow flexion. In the baseline and post-adaptation reaching tasks, the avatar’s hand was rendered invisible to prevent visual feedback of the limb. The image corresponds to the washout (WO) task condition under whih the avatar remained visible. **(b)** Articipants alternately touched two target objects at 4-s intervals in a repetitive task.

The reaching task was designed to evaluate motor performance. To assess changes in performance, this task was further divided into the *baseline reaching task* and the *post-adaptation reaching task* which were performed before and immediately after adaptation, respectively. In both tasks (Figure 4a), participants were instructed to reach the target object presented in the VR environment as quickly as possible by flexing the right elbow joint. The RBA condition was used; however, the avatar display was disabled (invisible) so that visual feedback of the upper limb did not influence the movement planning for the reaching action. Target objects were arranged along an arc such that they could be reached easily by flexing the elbow, i.e., their position was defined as the point reached by flexing the elbow by 60° from the initial hand position. The presentation timing of each target was randomized. Each set comprised 10–30 trials. The number of trials differed across participants because this is a pilot study, and the experimental protocol was iteratively refined during data collection. In the PAEB adaptation group and the RT control group, all participants performed 30 trials per set. In the PAEB nonadaptation group, two participants performed 20 trials, one participant performed 30 trials, and the remaining participants performed 10 trials. In the original RBA control group, two participants performed 30 trials and the remaining participants performed 10 trials.

**FIGURE 5:**
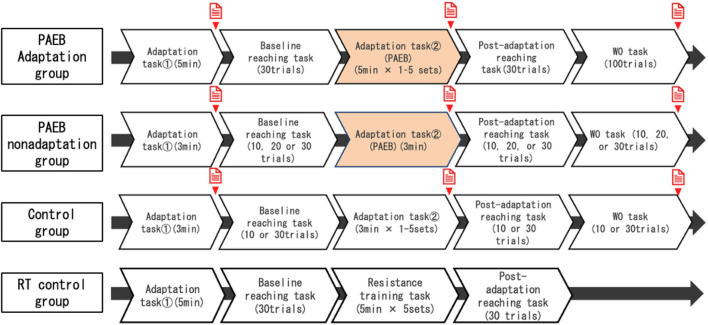
Experimental procedure for each group. Note: From top to bottom, the procedures for the PAEB adaptation, PAEB nonadaptation, control, and resistance training (RT) control groups are illustrated. Colored blocks indicate tasks performed using the PAEB system. During the adaptation task, the PAEB adaptation group completed up to five sets (5 min × 1–5) or until the adaptation criterion was met, whereas the PAEB nonadaptation group completed a single 3-min set. The control group performed all tasks using the RBA for up to five sets (3 min × 1–5) or until the adaptation criterion was met. The RT control group performed resistance training without visual avatar feedback for up to five sets (5 min × 1–5). Because the RT control group did not undergo avatar-based adaptation, a washout task was not included in their protocol. Questionnaires assessing the SoO and SoA were administered at the end of each task, as indicated by red icons.

**FIGURE 6:**
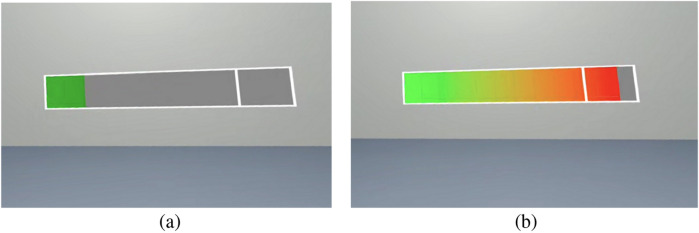
Visual force indicator used in the resistance training (RT) task. Note: In the RT control group, the participants were presented with a visual force indicator instead of an avatar. The indicator displayed a bar whose length and color changed in real time according to the exerted force. Examples of **(a)** low force output and **(b)** high force output are shown. The white frame represents the target force (more than 60% of maximum voluntary contraction).

The adaptation task (Figure 4b) was designed to facilitate adaptation to the avatar conditions. Participants alternately contacted two target objects positioned to the left and right at 4-s intervals while the avatar display (PAEB or RBA) was active. The targets were placed such that one was reached by flexing the elbow by 45° from the initial hand position and the other by extending the elbow by 30° . As illustrated in the experimental procedure (Figure 5), the task comprised two phases. The first phase (adaptation task②) served as a familiarization period using the RBA. The second phase (adaptation task) constituted the primary intervention, employing PAEB (adaptation and nonadaptation groups), RBA (control group), or a resistance training task (Figure 6) (RT control group). In the RT control group, to match the physical load of the PAEB condition, participants were provided with a visual force indicator on the HMD instead of an avatar. This indicator displayed a bar whose length and color changed in real time according to the exerted force, along with a target line corresponding to 60% of maximum voluntary contraction (MVC). The 60% MVC level was defined based on subjectively reported force exertion during the PAEB condition in the adaptation group. Participants were instructed to alternately perform isometric elbow flexion and relaxation in approximately 4-s cycles, matching the temporal pattern of the PAEB adaptation task. A trial was considered successful if the force output exceeded the target threshold for at least 0.5 s within each 4-s window. The duration of each set was 5 min for the PAEB adaptation and RT control groups and 3 min for the PAEB nonadaptation and control groups.

A washout (WO) task was performed as the final task to promote re-adaptation from PAEB back to RBA. The task content was similar to that of the reaching task; however, the avatar display was enabled and each target object disappeared upon contact. In this task, 10–100 trials were conducted per set. As in the reaching task, the number of trials varied across participants because protocol refinement and data collection were simultaneously performed. In the PAEB adaptation group, all participants performed 100 trials per set. In the PAEB nonadaptation group, two participants performed 20 trials, one participant performed 30 trials, and the remaining participants performed 10 trials. In the original RBA control group, two participants performed 30 trials and the remaining participants performed 10 trials.

Note that the WO task was not performed to evaluate motor performance. This was because unlike in the previous reaching task protocol, the avatar was displayed during the WO task; therefore, differences in visual feedback were expected to influence motor performance.

### 3.6 Procedures

Figure 5 shows the experimental procedures for each group. Participants in all groups first performed the adaptation task① using the RBA to adapt to the virtual environment.

Then, the baseline reaching task was performed to record the participants’ initial motor performance, after which the adaptation task② was performed. The PAEB adaptation and nonadaptation groups performed the task using the PAEB system, whereas the control group used the RBA, and the RT control group performed the resistance training task. The PAEB adaptation and control groups continued the adaptation task until they either met the adaptation completion criterion or completed five sets. The RT control group similarly continued until completing five sets (5 min per set). In contrast, the PAEB nonadaptation group performed the PAEB adaptation task for only one set that lasted 3 min.

The adaptation completion criterion for the PAEB adaptation and control groups was defined as the point at which the SoO/SoA questionnaire scores, which were assessed at the end of each set, reached the same values as those obtained after the adaptation task with the RBA.

A key methodological distinction was that, during the RBA experience, participants’ forearms were unfixed and could be freely flexed and extended. During the PAEB adaptation task, however, their forearms were fixed in place. Immediately after the adaptation task, a post-adaptation reaching task—with identical conditions to the baseline reaching task—was performed to record post-adaptation motor performance and evaluate the effects of adaptation.

Subsequently, the WO task was performed under the RBA display to assess re-adaptation to the RBA using the same questionnaire. A rest period of 1–2 min was provided between tasks to minimize fatigue.

Except RT control group, SoO and SoA were assessed using a seven-point Likert scale (1 = lowest, 7 = highest). Questionnaires were administered at three time points: after the adaptation task①, after the adaptation task②, and after the WO task. In the PAEB adaptation and control groups, additional SoO/SoA evaluations were performed at the end of each adaptation task② set.

The questionnaire items were as follows: for SoO, “Did you feel that the VR avatar’s arm was part of your own body?“. For SoA, “Did you feel that the movements of the VR avatar’s arm were caused by your own intention?”

### 3.7 Outcome measures

The primary outcome measure was the maximum angular velocity of right elbow flexion during the reaching task. The same measurement system and analysis procedure were applied in both the baseline and post-adaptation reaching tasks to ensure that the observed differences were only related to the presence or absence of adaptation. From the resulting angular velocity profile, the peak value within the movement interval of each trial was extracted as the primary evaluation value. This movement interval was defined as the period between the onset of target presentation and the moment when the fingertip reached the target object. The mean value of the peak angular velocity across trials was computed for each participant, and the rate of change before and after adaptation was calculated.

Changes in the SoO and SoA scores were also recorded as subjective outcome measures. To ensure data quality, trials meeting the predefined exclusion criteria such as tracking loss, missing target-contact detection, and obvious logging/measurement errors were excluded from the analysis.

### 3.8 Statistical analysis

#### 3.8.1 Analysis of subjective measures

The SoO and SoA scores (subjective measures) obtained at three time points (after the adaptation tasks with RBA and PAEB and after the WO task) were used (Okada and Mitake, 2024; Kalckert and Ehrsson, 2012; 2014; Hamasaki et al., 2015; Kubota et al., 2024). Experiencing altered bodily dynamics (PAEB) consistently reduced SoO and SoA scores; these scores recovered when visuomotor correspondence was restored (RBA). Therefore, one-tailed tests were performed for comparisons between these time points.

Specifically, Shapiro–Wilk tests were performed on the obtained scores for each group. If normality was confirmed across all groups, a paired t -test (one-tailed) was used; if normality was not confirmed in at least one group, the Wilcoxon signed-rank test (one-tailed) was used to determine the significance. Bonferroni correction was applied for performing multiple comparisons. The distributions of subjective scores were visualized using boxplots to assess skewness and potential outliers.

#### 3.8.2 Analysis of motor performance measures

For evaluating the motor performance, the maximum angular velocity of the elbow joint obtained in the baseline and post-adaptation reaching tasks was used. The authors hypothesized that adaptation to PAEB improved motor performance in a unidirectional manner; therefore, one-tailed tests were performed for evaluating motor performance. In addition, Shapiro–Wilk tests were performed for each condition to confirm normality. If normality was confirmed across all trials in a task, the representative value for each participant was defined as the mean of the maximum angular velocity across 10 trials in that task. If normality was not confirmed in any trial set, the representative value was defined as the median of the maximum angular velocity across 10 trials in that task. These representative values were used to evaluate changes in motor performance before and after adaptation. Specifically, for each participant, the representative maximum angular velocities determined before adaptation (baseline reaching task) and after adaptation (post-adaptation reaching task) were compared. If normality was confirmed across all groups, a paired t -test (one-tailed) was performed; if normality was not confirmed in at least one group, the Wilcoxon signed-rank test (one-tailed) was performed to determine the presence or absence of a significant difference within each group.

In addition, within-participant changes in maximum angular velocity were evaluated across groups. Depending on the normality determined using the Shapiro–Wilk test, either an unpaired t -test (one-tailed) or the Mann–Whitney U test (one-tailed) was performed.

#### 3.8.3 Selection of trial intervals for analysis

This section describes how differences in trial numbers across participants were addressed. To account for inconsistencies in total trial counts across early cohorts and to minimize the influence of task practice or familiarization, a standardized analytical approach was adopted. Motor learning is commonly described by a dual-process model comprising a rapid “fast adaptation” phase and a more gradual “slow adaptation” phase (Smith et al., 2006). Familiarization with the reaching task and VR environment corresponds to the fast adaptation phase, after which performance transitions to slow adaptation. During the initial 20–30 trials, performance gains driven by fast adaptation may lead to overestimation of improvement. To mitigate these effects and control for variation in task volume, motor performance was consistently evaluated using only the final 10 trials of the reaching task for all participants. This approach provides a more conservative estimate, as any transient aftereffects of adaptation may diminish during the later trials. To examine the validity of this trial segment selection, data from participants in the 30-trial subgroup (9 males; mean age 22.8±0.98 years) were analyzed by comparing the angular velocity data from the first 10 trials (1–10) and the last 10 trials (21–30). Shapiro-Wilk tests were applied to evaluate normality; if normality was confirmed, an unpaired t -test was used, and if normality was not confirmed, the Mann-Whitney U test was used.

#### 3.8.4 Evaluation metrics

The rate of change in the representative values for each participant was calculated using Equation 3. The effect size r is a descriptive statistic used for indicating the magnitude of adaptation effects: for t -tests, Equation 4 was used and for Mann–Whitney U tests and Wilcoxon signed-rank tests, Equation 5 was used (Fritz et al., 2012). $v_{\text{pre}}$ and $v_{\text{post}}$ denote the representative maximum angular velocity before and after adaptation, respectively. t denotes the test statistic, and df denotes the degrees of freedom for the test. In addition, Z represents the test statistic standardized via normal approximation for Mann–Whitney U tests. For Wilcoxon signed-rank tests, Z represents the test statistic W (rank sum) standardized based on the standard normal distribution. N represents the total number of data points used for comparisons. The interpretation of effect size r was the same as that for parametric tests, and the absolute value of r was interpreted using thresholds of approximately 0.10 (small), 0.30 (medium), and 0.50 (large) (Keskin and Aktas, 2013).

$$\text{rate of change}\left(\%\right)=\frac{v_{\text{post}}-v_{\text{pre}}}{v_{\text{pre}}}\times 100$$ (3)

$$r=\frac{t}{\sqrt{t^{2}+df}}$$ (4)

$$r=\frac{Z}{\sqrt{N}}$$ (5)

The significance level for all tests was set to α=0.05 , and all statistical analyses were implemented in Python using NumPy, pandas, matplotlib, and SciPy.

## 4 Results

To confirm baseline comparability, statistical analyses were conducted prior to the intervention. A one-way analysis of variance (ANOVA) revealed no significant differences among the four groups in age ( F=0.359 , p=0.783 ) or pre-intervention angular velocity ( F=0.071 , p=0.975 ). These results indicate the absence of systematic between-group differences at baseline.

### 4.1 Questionnaire results (SoO/SoA)

**FIGURE 7:**
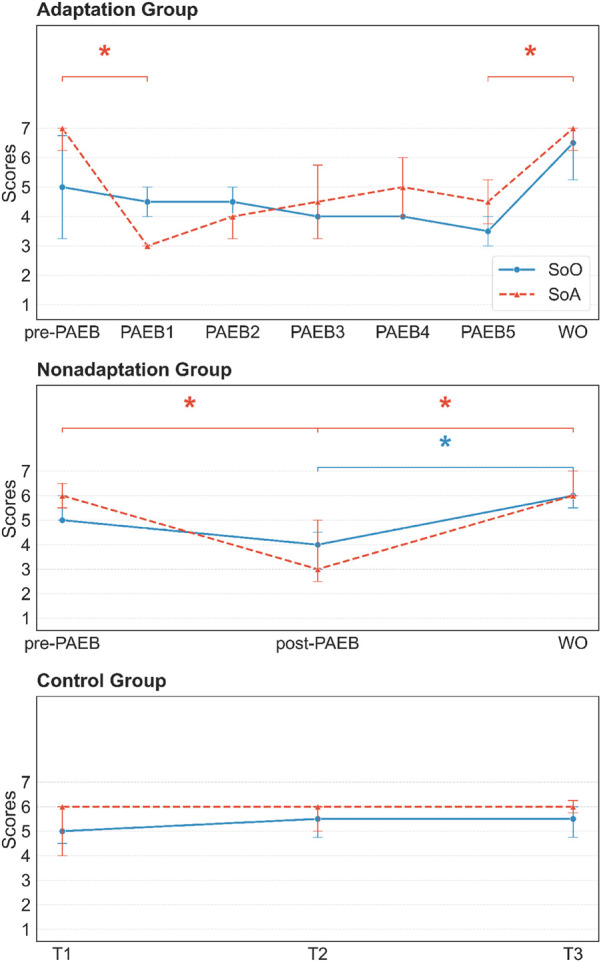
SoO and SoA scores for each group. Note: The PAEB adaptation group, PAEB nonadaptation group, and control group are shown. In the PAEB adaptation group, multiple adaptation tasks were performed using the PAEB; these tasks are labeled PAEB1, PAEB2, … PAEB(last) refers to the questionnaire results obtained at the final PAEB adaptation task for each participant. In the PAEB nonadaptation group, pre-PAEB refers to the score obtained before performing the adaptation task with PAEB and post-PAEB refers to the score obtained after. As the PAEB was not used in the control group, the task corresponding to pre-PAEB, post-PAEB, and the WO task are labeled T1, T2, and T3, respectively.

The SoO and SoA scores for each participant are shown in Tables 1–3. The time course of SoO and SoA differed across participants groups and measurement time points; the corresponding results are summarized in Figure 7. Shapiro–Wilk tests were performed for each phase in each group. As some groups did not satisfy the assumption of normality, changes in the median values were used for interpretation.

**TABLE 1:** PAEB adaptation group SoO and SoA.

Subject	Pre PAEB		PAEB1		PAEB2		PAEB3		PAEB4		PAEB5		WO	
-	SoO	SoA	SoO	SoA	SoO	SoA	SoO	SoA	SoO	SoA	SoO	SoA	SoO	SoA
A01	6	7	4	2	4	3	4	3	4	2	4	3	6	6
A02	7	7	5	3	5	4	4	3	a	a	a	a	7	7
A03	3	6	5	3	6	4	6	6	7	6	b	b	7	7
A04	4	6	4	3	4	4	3	5	4	5	3	5	5	6
A05	7	7	6	4	5	6	4	6	4	6	4	6	7	7
A06	3	7	3	3	3	3	3	4	3	4	3	4	3	7

^a^ A02 terminated the PAEB exposure after three sets due to equipment malfunction.

^b^ A03 completed four sets because the adaptation completion criterion was satisfied and therefore did not proceed to the fifth PAEB set.

**TABLE 2:** SoO and SoA scores of the PAEB nonadaptation group.

Subject	Pre PAEB		Post PAEB		WO	
-	SoO	SoA	SoO	SoA	SoO	SoA
B01	6	7	6	6	6	7
B02	5	4	3	2	5	6
B03	5	6	5	2	6	6
B04	5	5	4	3	6	6
B05	5	6	3	4	5	7
B06	7	7	3	6	6	7
B07	6	6	3	3	6	6

**TABLE 3:** Control group SoO and SoA.

Subject	T1^*^		T2^*^		T3^*^	
-	SoO	SoA	SoO	SoA	SoO	SoA
C01	5	6	5	6	5	6
C02	3	4	4	4	4	4
C03	6	6	6	6	6	6
C04	6	7	6	7	6	7
C05	2	4	3	5	3	5
C06	6	6	6	6	6	7
C07	5	6	6	6	6	6
C08	5	4	5	5	5	6

* In the control group, T1, T2, and T3 correspond to pre-PAEB, post-PAEB, and the WO task, respectively.

One participant in the PAEB group terminated the PAEB experience after three sets due to equipment malfunction. Another participant met the adaptation completion criterion after the fourth PAEB experience and did not proceed to a fifth set. All participants in the control group completed the adaptation task② in one set.

For statistical testing, two comparisons were made within each group before and after switching avatar conditions. Accordingly, the significance threshold was adjusted using the Bonferroni correction $\left(\alpha_{\text{adj}}=0.05/2=0.025\right)$ . Specifically, in the PAEB adaptation group, Wilcoxon signed-rank tests (one-tailed, significance level $\alpha_{\text{adj}}=0.025$ ) were performed for (i) pre-PAEB vs. PAEB1 and (ii) PAEB(last) vs. WO. In the PAEB nonadaptation group, tests were performed for (i) pre-PAEB vs. post-PAEB and (ii) post-PAEB vs. WO. In the control group, tests were performed for (i) T1 vs. T2 and (ii) T2 vs. T3. Here, PAEB(last) refers to the final set of the PAEB adaptation task for each participant in the PAEB adaptation group.

In the PAEB adaptation group, the SoO score decreased by 0.5 points although this change was not statistically significant. It also showed a gradual downward trend after repeated PAEB exposure. After the WO task, the SoO score increased by 3.0 points but was not statistically significant. In the PAEB nonadaptation group, no statistically significant difference was observed between pre-PAEB and post-PAEB, although a decrease of 1.0 points was noted. However, after the WO task, the SoO score significantly increased by 2.0 points. In the control group, no significant changes in the SoO score were observed throughout the experiment.

Note that in the PAEB adaptation group, a discrepancy was observed between the median trend of SoO scores (Figure 7, upper left panel) and the individual participant data (Table 1). The median remained low (3.5–4.5) throughout the PAEB experience (from PAEB1 to PAEB(last)). However, an analysis of the individual data revealed that participant A03, who met the adaptation completion criterion after the fourth PAEB session, showed a very high SoO score of 7. Thus, although the group median indicates a downward trend in the SoO score, successful adaptation may be associated with maintained or recovered SoO scores for individual participants.

In the PAEB adaptation group, the SoA score decreased by 4.0 points immediately after the first PAEB set (PAEB1) and this decrease was statistically significant (p<0.001) . However, with repeated PAEB exposure, the SoA score showed an upward trend; it increased by 1.5 points after the participant’s final PAEB session (PAEB(last)) compared with the first set. After RBA exposure in the WO task, the SoA score significantly increased by 2.5 points (p=0.010) . In the PAEB nonadaptation group, the SoA scores during RBA exposure (pre-PAEB and WO task) were 6.0 and 3.0 during PAEB experience, which was significantly lower (p=0.002) . In the control group, no changes in the SoA score were observed throughout the experiment.

### 4.2 Motor performance (maximum angular velocity)

The performance metric was defined as the maximum angular velocity during the reaching task in the experiment.

To examine whether the number of trials in the reaching task affected motor performance, angular velocity data from the first 10 trials (1–10) and the last 10 trials (21–30) in the 30-trial subgroup were compared. Results revealed no statistically significant difference ( p=0.984 , Mann–Whitney U , α=0.05 ).

**FIGURE 8:**
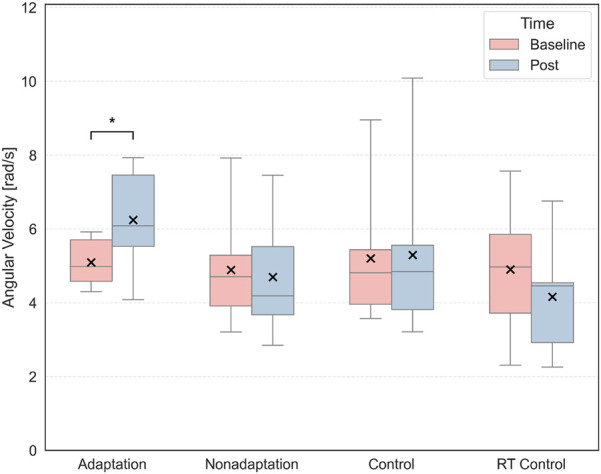
Maximum angular velocity for each group. Note: Angular velocity distributions in the baseline and post-adaptation reaching tasks are shown for all four groups (PAEB adaptation, PAEB nonadaptation, control, and RT control). * p<0.05 .

Within-group comparisons between the baseline and post-adaptation reaching tasks did not satisfy normality for any group. Therefore, medians were used in subsequent analyses. Figure 8 shows the distributions of maximum angular velocity across both tasks for each group. The rate of change in maximum angular velocity in the post-adaptation reaching task relative to the baseline reaching task is shown in Table 4. The PAEB adaptation group showed a 25.4% increase, the PAEB nonadaptation group showed a 5.86% decrease, the control group showed a 0.42% decrease, and the RT control group showed a 15.3% decrease in the maximum angular velocity. In other words, the PAEB adaptation group exhibited a markedly larger increase in angular velocity compared with the other three groups, while the RT control group showed the largest decrease. A comparison of the baseline and post-adaptation reaching tasks within each group using the Wilcoxon signed-rank test (one-tailed, significance level α=0.05 ) revealed a significant improvement in the angular velocity only in the PAEB adaptation group ( p=0.031 , effect size r=0.538 , indicating a pronounced effect). No significant improvement was observed in the remaining three groups (PAEB nonadaptation: p=0.711 , r=−0.149 ; control: p=0.527 , r=0.017 ; RT control: p=0.980 , r=−0.486 ).

**TABLE 4:** Change in maximum angular velocity for each group.

Group	Baseline [rad/s]	Post-adapt [rad/s]	Rate of ${\text{change}}^{†}$ [%]	p-value	Effect size r
PAEB adaptation	4.98	6.09	+ 25.4	0.031*	0.538
PAEB nonadaptation	4.70	4.18	− 5.86	0.711	− 0.149
Control	4.81	4.84	− 0.42	0.527	0.017
RT control	5.00	4.24	− 15.3	0.980	− 0.486

^†^The rate of change is the representative value of those calculated individually for each participant.

* p<0.05

For each participant in each group, the Shapiro–Wilk test was applied to the maximum angular velocity data from the baseline and post-adaptation reaching tasks to assess normality. Normality was not confirmed for some participants. Accordingly, Mann–Whitney U tests (one-tailed) were used to compare the baseline and post-adaptation reaching tasks for each participant. The results, along with the number of reaching trials performed by each participant, are shown in Table 5. In the PAEB adaptation group, the angular velocity significantly increased in five out of six participants. In contrast, no significant improvement was observed in any of the nine participants in the RT control group; instead, eight showed a decrease in maximum angular velocity after the intervention. Furthermore, only one out of seven participants in the PAEB nonadaptation group, and one out of eight participants in the RBA control group, exhibited a significant improvement. These findings indicate that improvements in motor performance were predominantly observed in the PAEB adaptation group.

**TABLE 5:** Results of the individual reaching task.

Subj.	Trials	p-val.
A01	30	0.948
A02	30	0.007*
A03	30	$<0.001^{*}$
A04	30	$<0.001^{*}$
A05	30	$<0.001^{*}$
A06	30	$<0.001^{*}$
B01	10	0.984
B02	10	0.781
B03	10	0.081
B04	10	0.920
B05	20	0.999
B06	20	0.023*
B07	30	0.740
C01	10	0.171
C02	10	0.214
C03	10	0.633
C04	10	0.688
C05	10	0.981
C06	10	0.013*
C07	30	0.515
C08	30	0.808
D01	30	0.715
D02	30	0.285
D03	30	0.485
D04	30	0.236
D05	30	0.894
D06	30	0.808
D07	30	0.999
D08	30	0.991
D09	30	0.999

Participants are categorized by the prefix of their ID (A: PAEB adaptation group, B: PAEB nonadaptation group, C: control group, and D: RT control group).

For participants who performed 20 or 30 trials, one-tailed significance testing was performed on the last 10 trials (significance level α=0.05 ).

* p<0.05

## 5 Discussion

In this study, it was hypothesized that recalibration of internal models through error learning optimizes motor control, leading to increased maximum angular velocity during reaching tasks. Accordingly, increases in maximum angular velocity are interpreted as indicative of motor improvement.

### 5.1 Interpretation of questionnaire results

In the control group, the avatar remained in the RBA condition throughout the experiment, such that the visuomotor correspondence was kept constant. Thus, no significant changes were observed in either SoO or SoA scores.

In contrast, in the PAEB adaptation and nonadaptation groups, the SoO and SoA showed a tendency to decrease during PAEB experience. In particular, the SoA score showed statistically significant decreases across both groups. In PAEB, the EMG-driven control introduced visuomotor mismatch involving delay and damping that could reduce the feeling of “Did you feel that the movements of the VR avatar’s arm were caused by your own intention?” Indeed, visuomotor asynchronies and delays have reduced the SoA score, consistent with the findings of this study (Haggard, 2017; Synofzik et al., 2008; Braun et al., 2018; Ciardo et al., 2020). Although PAEB was visually identical to RBA, this visual similarity was insufficient to maintain the SoO or SoA scores. The degradation in sensorimotor congruence due to altered dynamics likely contributed to the reduction in both the SoO and SoA scores. This finding was consistent with the model, wherein SoO depended on the integration of visual and somatosensory information as well as on visuomotor consistency (Kalckert and Ehrsson, 2012).

A noteworthy finding of this study is the subsequent trajectory in the PAEB adaptation group. The SoA showed a recovery trend with repeated exposure (PAEB1: 3.0 → PAEB(last): 4.5) that likely resulted from reduced prediction errors in the internal models via repetition. As such, the avatar’s behavior was perceived as aligned with the participant’s motor intent. As the SoA score strongly depended on the forward model that predicted the sensory consequences of motor commands, this score will decrease immediately after a drastic change in dynamics. It will then recover as re-adaptation progressed; this framework was supported by both theory and experimental evidence (Kilteni and Ehrsson, 2017; Gonzalez-Franco et al., 2019).

Herein, many participants did not experience full adaptation to the PAEB system due to the following factors. First, in both the PAEB adaptation group and nonadaptation groups, the participant’s physical arm performed isometric contractions while the avatar moved; this increased a mismatch between proprioceptive feedback and visual feedback. Second, the mismatch between the NMSS parameterization of PAEB dynamics and the actual dynamics of their own limb may have been too large for some participants. These factors possibly interfered with the alignment between predicted and observed sensory consequences required for SoA formation, making complete adaptation difficult.

### 5.2 Interpretation of motor performance

Regarding the mixture of early and late trial segments in the reaching task, the lack of a statistically significant difference between the first and last 10 trials indicated that the choice of analysis window did not strongly influence the corresponding results. In case of any influence, it would act conservatively with respect to the null hypothesis and would thus represent an evaluation under unfavorable conditions.

These findings indicated the PAEB adaptation group showed a tendency toward improvement in maximum angular velocity compared with the other groups. At the group level, the maximum angular velocity increased by 25.4% and a significant difference was confirmed within the group between the baseline and post-adaptation reaching tasks. An effect size r of 0.538 was obtained, suggesting that adaptation to PAEB significantly improved the motor performance.

A critical finding of this study is the stark contrast in motor performance outcomes between the PAEB adaptation and RT control groups. Despite both groups experiencing comparable physical load (isometric contractions with forearms immobilized) and proprioceptive conditions, five of six participants in the PAEB group exhibited significant speed improvements, whereas none in the RT control group did. Most of the RT participants exhibited a decrease in movement speed. This decline aligns with the authors’ previous findings, indicating that acute resistance training does not immediately improve peak velocity and that sustained isometric contractions induce muscle fatigue (Shi et al., 2022). Therefore, the improvement observed in the PAEB group is unlikely to be attributable to acute neuromuscular responses to physical load or to proprioceptive mismatch arising from forearm immobilization. The key distinction between groups was the visual input: only the PAEB group experienced a temporally delayed avatar. Within the internal model framework of motor control, this delay generates a sensory prediction error. To reduce this discrepancy, the central nervous system recalibrates internal models by increasing motor command gain to compensate for the perceived sluggishness of the limb. This recalibrated state may persist as an aftereffect during post-adaptation reaching with the real limb, resulting in faster movements. These findings suggest that the proposed PAEB system can enhance motor performance via error-driven recalibration of internal models, rather than through physical training effects. Furthermore, this comparison mitigates the concern that the post-intervention differences were merely due to varying levels of task practice or familiarization. The RT control group followed the same protocol as the PAEB adaptation group, sharing matched adaptation task durations (5 min/set) and reaching task trial counts (exactly 30 trials). Because a significant improvement was uniquely observed in the PAEB adaptation group, even when compared with this strictly matched RT control group, the authors suggest that the observed motor improvement is not attributable to differences in the task volume or initial familiarization but rather specifically driven by the PAEB intervention (i.e., recalibration of internal models).

For within-participant comparisons, five out of six participants in the PAEB adaptation group showed a significant increase in maximum angular velocity. This indicated that adaptation to the PAEB improved the motor performance of most participants. The one participant who did not show significant improvement may have experienced incomplete PAEB adaptation. This could be due to insufficient PAEB exposure time or to an excessively large mismatch between the participant’s physical limb dynamics and PAEB dynamics. Adaptation to avatars can vary substantially across individuals even under identical conditions, and the variability in PAEB adaptation was consistent with this general tendency (Gonzalez-Franco et al., 2019; Waltemate et al., 2018). No statistically significant improvement in maximum angular velocity was observed throughout the experiment in the PAEB nonadaptation and control groups, except for participant B06 in the PAEB nonadaptation group and participant C06 in the control group. Despite the short PAEB exposure, the SoA score for the participant B06 after PAEB was high at 6 (pre-experience: 7). This indicated that adaptation to the avatar progressed and may have contributed to improved angular velocity.

### 5.3 Limitations and future work

Several limitations of this study warrant consideration. First, the small sample size limited statistical power, particularly for within-group and between-group comparisons. In addition, the participant cohort consisted predominantly of young men, restricting the generalizability of the findings to broader populations and clinical groups. Future studies should therefore include more diverse cohorts, including patient populations. Second, due to the nature of the VR avatar intervention, physical blinding to the avatar conditions (PAEB vs. RBA) was not feasible. However, to minimize demand characteristics and unintended bias, participants were kept naïve to the specific study hypotheses and were not informed that the PAEB condition was expected to enhance movement speed. Third, interindividual differences in the mismatch between PAEB dynamics (as defined by NMSS parameters) and each participant’s actual limb dynamics may have contributed to variability in adaptation. Fourth, the study assumes that increases in maximum angular velocity reflect unidirectional motor improvement. This metric was considered appropriate because this study was a preliminary validation (pilot study) involving healthy subjects. However, as highlighted by the neurorehabilitation literature, an increase in movement speed does not necessarily equate to motor improvement in pathological individuals with impaired error learning processes, because they may exhibit compensatory or abnormal movement patterns. Therefore, the establishment of appropriate evaluation metrics tailored to specific pathological conditions and the use of two-tailed statistical testing where appropriate are necessary for future clinical applications. Furthermore, regarding the subjective evaluation of avatar embodiment, this study used single-item questions on a 7-point Likert scale to minimize the cognitive load on participants during repeated measurements between task sets (Hamasaki et al., 2015; Kubota et al., 2024). For future studies, the use of validated multi-item scales (e.g., standardized Embodiment Questionnaires) is recommended to ensure higher reliability and validity of the SoO and SoA assessments.

Finally, critical covariates, such as prior VR experience, baseline motor ability, and baseline EMG activity, were not systematically controlled. As these factors may influence both adaptation to the avatar and motor outcomes, future large-scale studies should collect these variables and account for them in the statistical analyses to more accurately isolate the effects of the PAEB intervention.

Therefore, future studies should increase the sample size, personalize PAEB parameter settings to minimize excessive mismatch, and determine the exposure threshold required for sufficient adaptation. In addition, the persistence of the observed effects remains unclear; whether medium- to long-term PAEB interventions yield sustained improvements in motor performance requires further investigation. To enhance the reliability and validity of the proposed system, the incorporation of objective, quantitative biomarkers, such as those used in visuomotor adaptation (VMA) studies, will be essential to complement subjective SoO and SoA measures (Suglia et al., 2023). Furthermore, exploring alternative EMG-based features for assessing error learning (Bicer et al., 2024) may further advance the framework. Finally, to improve patient engagement in clinical settings, future developments should consider integrating more immersive visual content, such as serious VR-based games.

## 6 Conclusion

In this study, how healthy participants adaptation to an avatar that “feels slower and harder to move than the real body” in the PAEB system influences motor performance was examined. Experimental results indicated that such an adaptation to the PAEB can improve the motor performance. Notably, although significant increases in maximum angular velocity were observed at the individual level in the PAEB adaptation group, no such improvements were found at the group level in the other groups, including the PAEB nonadaptation, RBA control, and RT control groups (the latter performed equivalent physical effort without visual avatar feedback). These findings suggest that enhancements in motor speed are more likely attributable to motor learning—specifically, internal model recalibration driven by visuomotor discrepancies—rather than to acute neuromuscular responses to physical load. Based on these findings, the number of participants will be increased, the protocol will be further refined, and the long-term persistence of these effects will be investigated in the future study.

## Data Availability

The raw data supporting the conclusions of this article will be made available by the authors, without undue reservation.
