# Correction: A pilot study on the effects of a physio-avatar EB experience on motor performance

**DOI:** 10.3389/fbioe.2026.1922428

**Published:** 2026-07-14

**Authors:** Ryoma Kojima, Kazuhiro Matsui, Kotaro Okada, Ruu Fujii, Keita Atsuumi, Yoshiki Mori, Hiroaki Hirai, Atsushi Nishikawa

**Affiliations:** 1 Graduate School of Engineering Science, The University of Osaka, Toyonaka, Japan; 2 Faculty of Information Science and Arts, Osaka Electro-Communication University, Shijonawate, Japan; 3 Graduate School of Information Sciences, Hiroshima City University, Hiroshima, Japan

**Keywords:** avatar embodiment, electromyography, internal models, motor performance, neurorehabilitation, sense of agency, sense of ownership, virtual reality

There was a mistake in Table 4 as published. The signs of the effect size values (*r*) for the PAEB adaptation and Control groups were incorrectly reported as negative values. The correct values are 0.538 and 0.017, respectively. The corrected Table 4 appears below.

**TABLE 4:** Change in maximum angular velocity for each group.

Group	Baseline [rad/s]	Post-adapt [rad/s]	Rate of change^†^ [%]	p-value	Effect size *r*
PAEB adaptation	4.98	6.09	+25.4	0.031*	0.538
PAEB nonadaptation	4.70	4.18	−5.86	0.711	−0.149
Control	4.81	4.84	−0.42	0.527	0.017
RT control	5.00	4.24	−15.3	0.980	−0.486

^†^ The rate of change is the representative value of those calculated individually for each participant.

* p<0.05 .

The original version of this article has been updated.

